# Endovascular management of traumatic pseudoaneurysms

**DOI:** 10.1186/s42155-020-00182-7

**Published:** 2020-11-27

**Authors:** Lauren Shreve, Maha Jarmakani, Hanna Javan, Ivan Babin, Kari Nelson, James Katrivesis, Michael Lekawa, Eric Kuncir, Dayantha Fernando, Nadine Abi-Jaoudeh

**Affiliations:** 1 Department of Radiological Sciences, University of California, Irvine, 101 The City Drive South, Rm 115 Rte 140, Orange, CA 92868 USA; 2 Department of Trauma Surgery, University of California, Irvine, Irvine, California, USA

**Keywords:** Trauma, Pseudoaneurysm, Endovascular treatment

## Abstract

**Background:**

Pseudoaneurysms (PAs) caused by traumatic injury to the arterial vasculature have a high risk of rupture, leading to life-threatening hemorrhage and mortality, requiring urgent treatment. The purpose of this study was to determine the technical and clinical outcomes of endovascular treatment of visceral and extremity traumatic pseudoaneurysms.

**Methods:**

Clinical data were retrospectively collected from all patients presenting for endovascular treatment of PAs between September 2012 and September 2018 at a single academic level one trauma center. Technical success was defined as successful treatment of the PA with no residual filling on post-embolization angiogram. Clinical success was defined as technical successful treatment with no rebleeding throughout the follow-up period and no reintervention for the PA.

**Results:**

Thirty-five patients (10F/25M), average age (± stdev) 41.7 ± 20.1 years, presented with PAs secondary to blunt (*n* = 31) or penetrating (*n* = 4) trauma. Time from trauma to intervention ranged from 2 h - 75 days (median: 4.4 h, IQR: 3.5–17.1 h) with 27 (77%) of PAs identified and treated within 24 h of trauma. Average hospitalization was 13.78 ± 13.4 days. Ten patients underwent surgery prior to intervention. PA number per patient ranged from 1 to 5 (multiple diffuse). PAs were located on the splenic (*n* = 12, 34.3%), pelvic (*n* = 11, 31.4%), hepatic (*n* = 9, 25.7%), upper extremity/axilla (*n* = 2, 5.7%), and renal arteries (*n* = 1, 2.9%). Technical success was 85.7%. Clinical success was 71.4%, for technical failure (*n* = 5), repeat embolization (*n* = 1) or post-IR surgical intervention (*n* = 4). There was no PA rebleeding or reintervention for any patient after discharge over the reported follow-up periods. Three patients died during the trauma hospitalization for reasons unrelated to the PAs.

**Conclusions:**

Endovascular treatment of traumatic visceral and extremity PAs is efficacious with minimal complication rates and low reintervention requirements.

## Background

Pseudoaneurysms (PA) are contained disruptions of the vascular wall leading to turbulent blood flow and hematoma formation (Keeling et al. 2009). Unlike true aneurysms, PAs are contained by only one or two of the three normal arterial wall layers, typically the adventitia alone (Hemp and Sabri 2015). Arterial damage leading to the formation of PAs may be caused by trauma, inflammation, infection, or iatrogenic sources (Keeling et al. 2009; Hemp and Sabri 2015; McDermott et al. 1994). PAs secondary to trauma may be related to either blunt or penetrating arterial trauma. While many PAs may be asymptomatic, they can present with swelling, pain, or mass effect (Saad et al. 2005). Due to the weakened arterial walls, PAs have a high risk of growth and rupture, leading to life-threatening hemorrhage and mortality, requiring urgent treatment.

In the past, identified PAs were treated surgically with arterial reconstruction, ligation, or end organ resection (Keeling et al. 2009; Batagini et al. 2016; Sachdev et al. 2006). Given the unstable nature of these patients, morbidity and mortality rates could be as high as 50% for surgical repair (Mandel et al. 1987; Stabile et al. 1983). Endovascular treatment of PAs is now increasingly common with advancements in medical management, imaging, endovascular techniques, and available embolic agents, however, minimal data currently exists reporting these outcomes (Saad et al. 2005; Sclafani et al. 1991; Shanmuganathan et al. 2000). While multiple case series have investigated the endovascular treatment of PAs, these cover PAs of mixed etiologies (e.g. trauma, infection, iatrogenic), combine outcomes of injury type (e.g. blush/active bleeding, true aneurysms, and PAs), or report a single organ alone (McDermott et al. 1994; Batagini et al. 2016; Sachdev et al. 2006; D’Souza et al. 2007; Guillon et al. 2003; Loffroy et al. 2008; Tessier et al. 2003; Tulsyan et al. 2007; Venkatesh et al. 2005; Zarzaur et al. 2017). Two case series with specific outcomes of traumatic pseudoaneurysms have been reported, however, assessing specifically the liver (*n* = 7) in patients failing initial conservative management, or the kidney (*n* = 3) (Osterballe et al. 2014; Yamacake et al. 2013). The purpose of this study was to determine the technical and clinical outcomes of endovascular treatment of visceral and extremity traumatic pseudoaneurysms.

## Methods

Institutional board review was obtained for this study and consent was waived. Clinical data were retrospectively collected from all patients presenting for treatment of PAs between September 2012 and September 2018 at a single academic level one trauma center. Inclusion criteria were patients who had sustained trauma, who had PA(s), and were treated by endovascular approach with interventional radiology (IR). Exclusion criteria were PA(s) of non-traumatic origin, contrast extravasation on CT/angiogram without a defined pseudoaneurysm, diffuse PA(s) in the head/neck or coronary vessels, parenchymal hemorrhage, or laceration, as defined by Zarzaur et al. (Zarzaur et al. 2017). PAs identified or treated over 3 months after trauma were also excluded to best ensure a traumatic etiology. Patient records were reviewed for demographic information, trauma type (blunt vs. penetrating), transfusion requirements pre- and post-treatment, PA(s) location, procedural technique, additional interventions, and clinical outcomes, including: length of hospitalization, ICU requirements, morbidity, and mortality. All available patient data were utilized for the follow-up period.

### Procedural technique

All patients presenting for trauma (blunt or localized) underwent primary assessment followed by contrast enhanced computed tomography (CT) for vascular evaluation. Imaging indicative or suspicious for arterial disruption was referred to IR for intervention. All endovascular interventions were performed by Interventional Radiologists in dedicated endovascular suites. Informed or emergency consent was obtained and sedation was provided based on individual patient assessment and needs, ranging from moderate sedation to general anesthesia. All patients were placed supine, with both groins prepped and draped.

Percutaneous access was obtained under ultrasound guidance using a standard micropuncture set. Seldinger technique was used to upsize and a 5 French (Fr) vascular sheath was inserted in the right or left common femoral arteries. A standard 5 Fr catheter (e.g. Cobra 2 (Merit Medical, Salt Lake City, UT, USA), Roberts Uterine Catheter (RUC) (Cook, Bloomington, IN, USA) or SOS (Angiodynamics, Latham, NY, USA)) depending on patient anatomy and PA location was subsequently used to selectively catheterize the culprit artery according to CTA imaging and angiography was then performed. Co-axial technique with a microcatheter was used to sub-selectively catheterize the PA artery. Treatment was then performed with Gelfoam slurry (Pfizer, New York, NY, USA), coil embolization, or both. Following treatment, post-embolization angiography was performed. At the completion of the procedure, the 5 Fr catheter and femoral vascular sheath were removed and hemostasis obtained via a closure device or manual pressure. Sterile dressing was applied.

Technical success was defined as successful treatment of the PA with no residual filling on post-embolization angiogram (Guillon et al. 2003; Loffroy et al. 2008). Clinical success was defined as technically successful treatment with no rebleeding throughout the follow-up period and no reintervention for the PA (Loffroy et al. 2008). Complications were defined in accordance with the Society of Interventional Radiology (SIR) adverse event (AE) part A classification (Khalilzadeh et al. 2017). Statistical analyses and Kaplan-Meier curves were performed with SPSS Statistical Software (Version 25, IBM, Armonk, NY, USA).

## Results

### Patient characteristics

One hundred four patients were treated with Interventional Radiology (IR) by endovascular management for PAs over the six-year study period. Thirty-five patients fulfilled the inclusion/exclusion criteria, with the majority excluded for PAs of non-traumatic etiology (e.g. pancreatitis, malignancy, or iatrogenic). Follow-up data after trauma hospitalization ranged from 0 days-4.4 yrs. (median: 44 days; IQR: 2.3–205.5 days).

All 35 patients (10F/25M), average age (± stdev) 41.7 ± 20.1 years, presenting with blunt (*n* = 31) or penetrating (*n* = 4) trauma, underwent endovascular treatment with IR. Time from trauma to IR intervention ranged from 2 h - 75 days (median: 4.4 h, IQR: 3.5–17.1 h) with 27 (77%) of PAs identified and treated within 24 h of trauma. Average hospitalization was 13.78 ± 13.4 days. Average ICU stay was 9.2 ± 10.11 days. Ten patients underwent a surgical procedure prior to IR intervention. Seven of the surgical treatments were performed near the site of PA location. Four patients underwent abdominal exploratory laparotomy, followed by treatment of liver or gastroepiploic PAs. Three patients underwent pelvic exploration or orthopedic pelvic/femur fracture repair and were subsequently treated for iliac or femoral PAs. Four of seven patients underwent surgical and IR intervention the same day, ranging from 58 min (direct transfer from the OR) to 7.3 h.

### Pseudoaneurysm characteristics

**Fig. 1:**
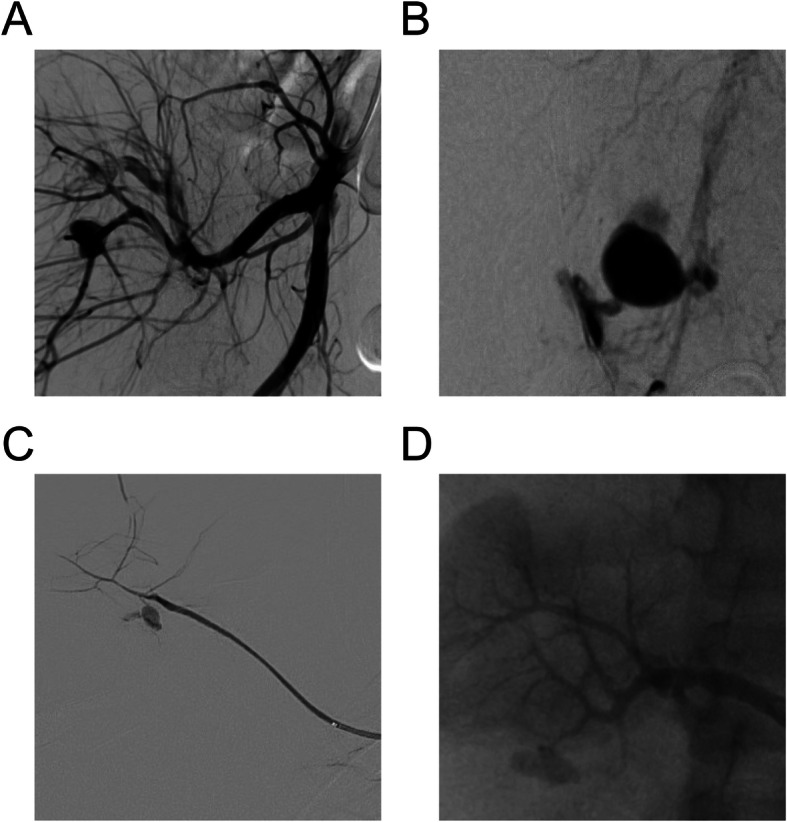
Angiographic image examples of traumatic pseudoaneurysms of the **a**) right superior gluteal artery, **b**) left internal pudendal artery, **c**) accessory right suprascapular artery, and **d**) right renal artery

PA number per patient ranged from 1 to 5 (multiple diffuse), with the largest measuring 4.3 × 3.1 cm. PAs were located on the splenic artery (*n* = 12, 34.3%), pelvic artery (including iliac, femoral, gluteal and pudendal vessels) (*n* = 11, 31.4%), hepatic (*n* = 9, 25.7%), upper extremity/axilla (*n* = 2, 5.7%), and renal arteries (*n* = 1, 2.9%) (Fig. 1).

### Procedural outcomes

**Fig. 2:**
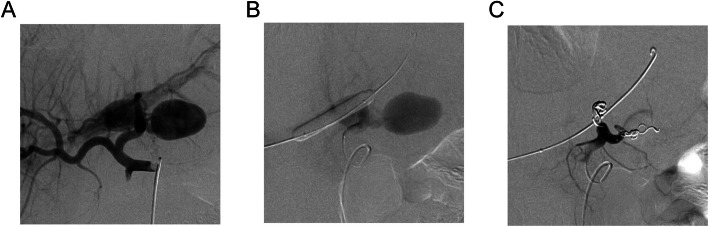
**a**) Angiographic image of a traumatic pseudoaneurysm with associated portal-venous fistula, treated with **b**) simultaneous portal venous access and balloon occlusion, followed by **c**) coil embolization of the pseudoaneurysm and fistula

Technical success was 30/35 (85.7%). Two patients were not treated based on angiographic and clinical data after discussion between IR and trauma surgeons. Three patients were not treated due to location of the PA. One case was a pediatric patient with an axillary wide-neck PA preventing coil embolization or stent placement, the other two were unable to be treated due to an inability to access the PA or treat safely from a distal location without risk of non-target embolization. Of these five patients, four underwent follow up surgical intervention with treatment of the traumatic region and PA. The remaining 30 patients were treated with coils (*n* = 6) (Azur Coils: Terumo, Shibya, Tokyo, Japan; Tornado Coils: Cook Medical, Bloomington, IN, USA; Nester Coils: Cook Medical, Bloomington, IN, USA; Concerto Coils: Medtronic, Minneapolis, MN, USA), gelfoam slurry (*n* = 19) (Pfizer, New York, NY, USA) or both (*n* = 5). Three patients (8.6%) had PAs with associated arteriovenous or portal-venous fistulas (Fig. 2). All were successfully treated with coil embolization.

**Fig. 3:**
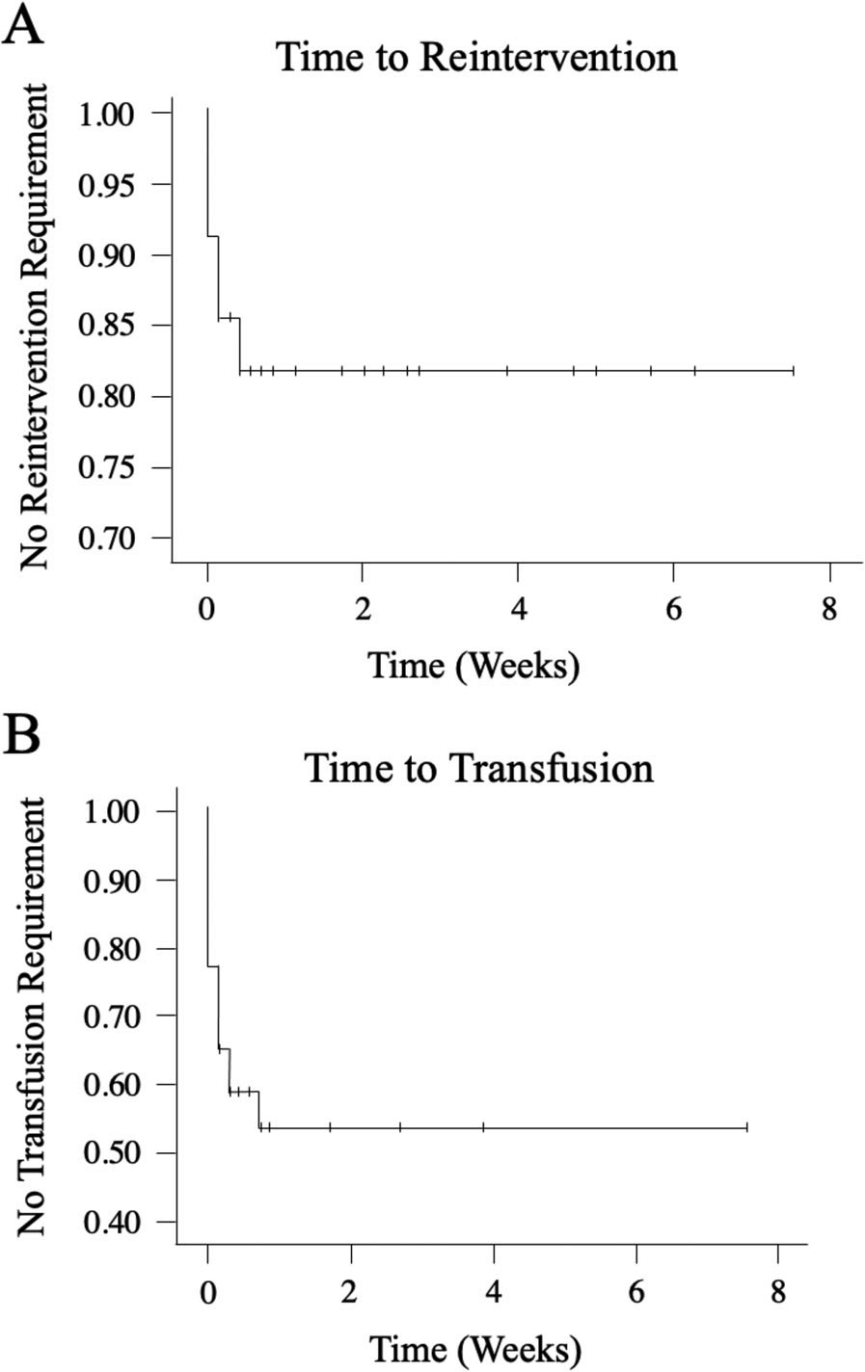
Kaplan Meier curves showing time to **a**) reintervention or **b**) transfusion after endovascular intervention for traumatic pseudoaneurysms

Clinical success was 25/35 (71.4%). In patients who were treated, success was 25/30 (82.9%). In all 5 cases of clinical failure, repeat interventions occurred within 3 days of initial treatment and within the same hospitalization (Fig. 3). One patient with an iliac PA treated initially within 24 h of trauma with gelfoam only underwent repeat embolization with gelfoam due to persistent bleeding. The remaining 4 clinical failures required surgical intervention. Three patients underwent splenectomy for persistent splenic PAs. Two of the three patients were treated with gelfoam only while the third underwent embolization with gelfoam and coils. The 4th patient had a gastroepiploic PA treated with Gelfoam as well. That patient required re-exploration due to persistent bleeding with hematoma evacuation.

There was no PA rebleeding or reintervention for PA treatment for any patient after discharge over the reported follow-up periods. Complications occurred in two patients (5.7%). The first complication was classified as an SIR mild AE, consisting of a common femoral PA that resolved without therapy. The second complication was classified as an SIR moderate AE, consisting of pedal punctate arterial emboli that resolved with a heparin drip (moderate escalation of care), without sequelae. Fifteen patients (42.8%) required red blood cell transfusions post-embolization, all occurred within 5 days of IR-intervention (Fig. 3).

Three patients died during the trauma hospitalization for reasons unrelated to the PA. Causes of death included (1) cerebral herniation leading to cardiac arrest, (2) abdominal compression syndrome followed by surgical exploratory laparotomy and profuse coagulopathy bleeding from abdominal wall collaterals, and (3) hypoxemia followed by palliative extubation per the patient’s family’s wishes. All three patients had technically and clinically successful treatment of their PAs.

## Discussion

PAs secondary to trauma place patients at high risk of hemorrhage and subsequent mortality. Advancements in diagnostic and endovascular technologies have improved the identification and management of PAs in trauma patients (Keeling et al. 2009). However, minimal literature evaluating the clinical outcomes of traumatic PA endovascular treatment currently exists. Case series evaluating PAs of mixed etiologies (e.g. infection, iatrogenic, trauma, inflammation) report clinical/technical outcomes of 85.7% - 100%, 71.4% - 100%, respectively (D’Souza et al. 2007; Tessier et al. 2003; Venkatesh et al. 2005; Zarzaur et al. 2017). Two organ specific traumatic PA case series have been reported accounting for PAs in the liver (*n* = 7) after failing conservative treatment, and kidney (*n* = 3) (Osterballe et al. 2014; Yamacake et al. 2013). When evaluating across all possible studies for traumatic PA outcomes only, technical and clinical success rates were reported ranging from 50 to 100%, and 86.36–100%, respectively (Loffroy et al. 2008; Zarzaur et al. 2017; Osterballe et al. 2014; Yamacake et al. 2013).

Here we present one of the largest case studies of endovascular management of traumatic PAs to date, covering a range of PAs locations both in the viscera and extremities. Our technical success and clinical success rates were 85.7% and 71.4%, respectively, which are similar to the previously reported mixed etiology/type and organ-specific series in the literature. Our cohort experienced no severe, life threatening, or disabling complications of treatment and nor any significant morbidities or mortalities secondary to endovascular treatment or PA re-bleeding.

Endovascular treatment of PAs has become increasingly common over surgical management (Sclafani et al. 1991; Berceli 2005). Previous studies comparing endovascular versus surgical treatment have reported shorter procedural times, less blood loss, reduced transfusion requirements, and shorter lengths of stay for the treatment of both true aneurysms and PAs (Batagini et al. 2016; Sachdev et al. 2006). These studies also found no difference between surgery and endovascular treatment in technical or clinical success, major complication rate, or overall survival (Batagini et al. 2016; Sachdev et al. 2006). Further, additional studies have pointed out the advantages of endovascular management in having lower risk than traditional surgical approaches while allowing for precise localization of the PA as well as assessment of the collateral vasculature (Hemp and Sabri 2015; Clark et al. 2017; Salam et al. 1992). Recent randomized controlled data has additionally shown that prophylactic splenic artery embolization after high risk splenic trauma results in fewer splenic PAs, secondary embolizations, and shorter hospitalizations, without increasing complication rates or compromising future splenic viability compared to surveillance (Arvieux et al. 2020).

However, traumatic patients in particular are difficult to manage, typically presenting with a plethora of injuries in addition to vascular trauma and PA formation. Therefore, it may be difficult to discern outcomes of PA management from overall morbidity and treatment requirements. In this cohort, 42.8% required post-IR embolization transfusions. This requirement may be related to blood loss secondary to the PA or related to the overall trauma. In addition, three patients with splenic PAs required splenectomy after successful IR treatment of a splenic PA, however, all had extensive splenic injury (grade ≥ 3). Previous studies have indicated that higher-grade splenic injuries have a higher likelihood of splenectomy (Zarzaur et al. 2017). It is worth noting, that our institution does not perform proximal splenic artery coil embolization for splenic hemorrhage control, which is an option to control diffuse splenic parenchymal hemorrhage in high grade splenic injuries.

This study was limited by retrospectively collected data with a small number of available patients. Follow-up data was therefore limited to available recorded information. Trauma patients are typically transported to the nearest hospital after an event and in many cases choose to follow-up with other physicians or health care institutions, limiting availability of information. For the purpose of ensuring a traumatic etiology, PAs treated more than 3 months post trauma were excluded, however, this approach may have conservatively eliminated PAs with further delayed presentations. Additionally, seven patients in this study underwent surgical intervention near the subsequently treated PA site, prior to IR PA embolization. Three of these cases were performed over a day apart, potentially confounding the etiology of true traumatic PAs with potentially iatrogenic PAs. Finally, as mentioned previously, the trauma patient cohort typically has significant injuries relating to the overall trauma, which may confound the clinical outcomes relating to PA treatment in isolation. Further randomized controlled data of endovascular management of traumatic PAs will be crucial in better understanding the clinical course of traumatic PAs.

## Conclusion

Endovascular management of PAs in patients with traumatic injuries can be effectively instituted into the trauma workflow. The endovascular treatment of traumatic visceral and extremity PAs is efficacious with minimal complication rates and low reintervention requirements.

## Data Availability

The datasets generated and analyzed during the current study are not publicly available due to containment of information that could compromise participant privacy/consent but may be available from the corresponding author in deidentified formats upon reasonable request.
